# Effects of cage vs. net-floor mixed rearing system on goose spleen histomorphology and gene expression profiles

**DOI:** 10.3389/fvets.2024.1335152

**Published:** 2024-02-13

**Authors:** Qingliang Chen, Yang Song, Zhiyu He, Guang Yang, Junqi Wang, Xiaopeng Li, Wanxia Wang, Xin Yuan, Jiwei Hu, Hua He, Liang Li, Jiwen Wang, Shenqiang Hu

**Affiliations:** ^1^Farm Animal Genetic Resources Exploration and Innovation Key Laboratory of Sichuan Province, Sichuan Agricultural University, Chengdu, China; ^2^Key Laboratory of Livestock and Poultry Multi-omics, Ministry of Agriculture and Rural Affairs, College of Animal Science and Technology, Sichuan Agricultural University, Chengdu, China; ^3^State Key Laboratory of Livestock and Poultry Breeding, College of Animal Science and Technology, Sichuan Agricultural University, Chengdu, China; ^4^Department of Animal Production, General Station of Animal Husbandry of Sichuan Province, Chengdu, Sichuan, China

**Keywords:** goose, dryland rearing system, spleen, histomorphology, gene expression

## Abstract

Due to the demands for both environmental protection and modernization of the goose industry in China, the traditional goose waterside rearing systems have been gradually transitioning to the modern intensive dryland rearing ones, such as the net-floor mixed rearing system (MRS) and cage rearing system (CRS). However, the goose immune responses to different dryland rearing systems remain poorly understood. This study aimed to investigate and compare the age-dependent effects of MRS and CRS on the splenic histomorphological characteristics and immune-related genes expression profiles among three economically important goose breeds, including Sichuan White goose (SW), Gang goose (GE), and Landes goose (LD). Morphological analysis revealed that the splenic weight and organ index of SW were higher under CRS than under MRS (*p* < 0.05). Histological observations showed that for SW and LD, the splenic corpuscle diameter and area as well as trabecular artery diameter were larger under MRS than under CRS at 30 or 43 weeks of age (*p* < 0.05), while the splenic red pulp area of GE was larger under CRS than under MRS at 43 weeks of age (*p* < 0.05). Besides, at 43 weeks of age, higher mRNA expression levels of *NGF*, *SPI1*, and *VEGFA* in spleens of SW were observed under MRS than under CRS (*p* < 0.05), while higher levels of *HSPA2* and *NGF* in spleens of LD were observed under MRS than under CRS (*p* < 0.05). For GE, there were higher mRNA expression levels of *MYD88* in spleens under CRS at 30 weeks of age (*p* < 0.05). Moreover, our correlation analysis showed that there appeared to be more pronounced positive associations between the splenic histological parameters and expression levels of several key immune-related genes under MRS than under CRS. Therefore, it is speculated that the geese reared under MRS might exhibit enhanced immune functions than those under CRS, particularly for SW and LD. Although these phenotypic differences are assumed to be associated with the age-dependent differential expression profiles of *HSPA2*, *MYD88*, *NGF*, *SPI1*, and *VEGFA* in the goose spleen, the underlying regulatory mechanisms await further investigations.

## 1 Introduction

China is the world’s largest producer and consumer of broiler geese, and the consumption of goose products has been rising year by year (1). Also, China is rich in goose genetic resources, having 31 indigenous goose breeds and several imported European goose breeds. All Chinese indigenous goose breeds, except for the Yili goose, are derived from swan goose (*Anser cygnoides*), while Yili goose and European domestic breeds are derived from greylag goose (*Anser anser*) (2). In recent years, due to the demands for both environmental protection and modernization of the goose industry in China, the goose rearing systems have been gradually transitioning from the traditional waterside rearing ones to the modern intensive dryland rearing ones, such as the floor rearing system (FRS), net rearing system (NRS), net-floor mixed rearing system (MRS), and cage rearing system (CRS) (3).

There is emerging evidence that the production and health of poultry were significantly affected by the rearing system. It has been demonstrated that birds reared under indoor and outdoor systems showed different immune responses. The outdoor environment exposed birds to a wider range of microorganisms and other antigens, which could stimulate their immune systems to develop rapidly and hence showed stronger immune responses (4, 5). The indoor dryland rearing systems such as MRS and CRS, which are more conducive to controlling the housing conditions by realizing the separation of manure, can greatly improve poultry production efficiency and health. A previous study reported that Desi broiler chickens reared under CRS exhibited better growth performance (such as feed conversion ratio, body weight, and breast muscle rate) than those under FRS (6). Similarly, compared to FRS, Chaohu ducks reared under NRS exhibited better growth performance, such as body weight and average daily gain (7). By comparison, less is known about the effects of different rearing systems on goose production and health. It was shown that the geese raised in pens equipped with a swimming pool showed a lower body weight, feed intake, and feed conversion ratio compared to those under FRS (8). Another study reported that the Yangzhou geese reared under CRS showed a higher average daily gain and body weight than those under FRS (3). Besides, the rearing system was reported to significantly alter the goose ileal histomorphological characteristics and cecal microbial composition, which might affect their physiological functions and production performance (9). Nevertheless, to date, the goose immune responses to different dryland rearing systems remain poorly understood.

The spleen, serving as a major immune organ in poultry, plays a crucial role in both the humoral and cellular immune responses by supporting the generation, maturation, and storage of lymphocytes (10). The expression levels of key immune-related genes in the poultry spleen are commonly utilized as key indicators of their immune responses (11). It has been shown that the rearing system is involved in regulating the expression of immune-related genes to modulate poultry immune functions. For instance, the broiler chickens reared under FRS exhibited higher mRNA expression levels of interleukin-1β and interferon-γ in the ileum than those under CRS (12). Compared to FRS, the NRS increased the serum levels of interleukin-1β, interferon-γ, interleukin-4, and immunoglobulins in meat ducks, consequently enhancing the ducks’ immune functions (13). Compared with the indoor rearing system, the mRNA expression levels of toll like receptor 7 (*TLR7*) were significantly higher in the bursa, lung, duodenum, ileum, and cecum of the free-range reared ducks (14). Also, expression of several key immune-related genes, including toll like receptor 2 (*TLR2*), toll like receptor 4 (*TLR4*), nuclear factor kappa B subunit 1 (*NF-κB1*), and interleukin 6 (*IL-6*), significantly increased in spleens of broiler chickens reared under FRS than those under CRS (15). These results altogether suggested that the rearing system may affect poultry splenic development and immune responses by modulating the activity of the TLR2/4, NF-κB, and MAPK signaling pathways. Of particular note, the unique NF-κB signaling pathway has been well demonstrated to play a central role in mediating the cellular immune responses to external stimuli (16, 17). Therefore, it is also important to explore the molecular mechanism by which the rearing system exerts the age-dependent effects on the goose splenic development and immune functions by examining the expression profiles of these key immune-related genes.

Considering that both the two Chinese indigenous goose breeds (Sichuan White goose, SW and Gang goose, GE) and one imported European goose breed (Landes goose, LD) have been extensively raised in China due to their remarkable traits and significant economic values, it is important to compare the impacts of different dryland rearing systems on the immune responses of these three goose breeds. The present study aimed to firstly compare the age-dependent effects of MRS and CRS on the splenic histomorphological characteristics among three goose breeds and to subsequently identify the key genes responsible for the age-dependent variations in the splenic immune responses under different rearing systems among three goose breeds. These results are expected to provide a theoretical basis for enhancing goose immune functions under dryland rearing systems.

## 2 Materials and methods

### 2.1 Experimental animals

Three medium-sized goose breeds including SW, GE, and LD were used in this study and were provided by the Waterfowl Breeding Experimental Farm of Sichuan Agricultural University (Ya’an, Sichuan, China). A total of 100 one-day-old male geese from each of these three goose breeds were hatched from the same batch and fed under the same environmental conditions during the period from 0 to 10 weeks of age. Subsequently, healthy geese from each breed were equally divided into two groups (MRS and CRS), and were provided free access to feed and water until sample collection. The geese under CRS were raised in individual cages with the dimensions of length × width ×height: 0.55 m × 0.37 m × 0.7 m, at a height of 1.5 m above the ground level. The geese under MRS were reared in an indoor area with the dimensions of length × width: 6 m × 13 m, which consisted of a 60 m^2^ plastic nets at a height of 1 m above the ground level and an 18 m^2^ fermentation bed. The daily lighting schedule for geese under both rearing systems is 16 h on and 8 h off, with lights on at 08:00 a.m.

### 2.2 Sample collection

At both 30 (reaching sexual maturity) and 43 (reaching body maturity) weeks of age, the body weights were measured after 12 h of fasting, and 8 male geese with similar body weights from each goose breed were randomly selected from CRS and MRS, respectively. All selected experimental geese (*n* = 8 per week per breed per rearing system) were euthanized by inhaling carbon dioxide, followed by cervical dislocation. After slaughter, the spleen and thymus were quickly collected and weighed. The spleen/thymus organ index was calculated using the following formula: spleen/thymus organ index (%) = (spleen/thymus organ weight (g)/body weight (kg)) × 100%. For each group, half of the spleens from each breed at each sampling point (*n* = 4) were used for histological examination, while the remaining (*n* = 4) were rapidly frozen in liquid nitrogen and finally stored at −80°C until RNA extraction.

### 2.3 Histological observation

The freshly-collected goose spleens were firstly fixed with 4% formaldehyde at room temperature for 72 h, then were dehydrated through a series of different concentrations of ethanol, and finally were transferred to xylene and embedded in paraffin-wax (18). Finally, each sample was cut into 5-μm thick slices, which were stained with hematoxylin and eosin (H&E). These stained slices were observed and photographed using a digital trinocular camera microscope BA410-Digital (Motic China Group Co. Ltd., Xiamen, China). According to the methods described in several previous studies (19–21), the goose splenic histological paraments were analyzed using Image-Pro Plus 6.0 software (National Institutes of Health, Bethesda, MD).

In detail, the goose splenic red pulp area (RPA) was observed by measuring the area of interest (AOI) of the area covered by red pulp. The splenic corpuscle area (ALA) was observed by measuring the AOI of splenic corpuscle. The splenic corpuscle diameter (ALD) was calculated by measuring the average diameter of the AOI of splenic corpuscle. The splenic trabecula area (TLA) was observed by measuring the AOI of splenic trabecula. The trabecular artery diameter (TAD) was calculated by measuring the average diameter the AOI of trabecular artery. The central artery diameter (CAD) was calculated by measuring the average diameter the AOI of the central artery. The mean diameter or area of each splenic histological paraments was calculated as the mean of the observations of all examined individuals per experimental group.

### 2.4 RNA extraction and quantitative real-time reverse transcription PCR (RT-qPCR)

Total RNA was extracted from the spleen tissues by Trizol reagent (Invitrogen, Massachusetts, CA, United States) following the manufacturer’s instruction. The RNA quality was assessed by a NanoDrop 2000 spectrophotometer (Thermo Fisher Scientific, Waltham, MA) and electrophoresis on a 1.5% agarose gel. Approximately 1 μg of total RNA from each sample was reverse transcribed into cDNA by HiScript^®^ RT SuperMix for qPCR (+gDNA wiper; Vazyme, Nanjing, China) according to the manufacturer’s instruction. Reactions of RT-qPCR were performed on the Bio-Rad CFX96 Real-time PCR Detection System (Bio-Rad, Hercules, CA, United States) using 2 × UDG Fast SYBR Green qPCR Master Mix (Cofitt, Chengdu, China). The reaction system was performed in a total volume of 20 μL, including 10 μL of 2 × UDG Fast SYBR Green qPCR Master Mix (Cofitt, Chengdu, China), 0.4 μL of each primer, 0.4 μL of cDNA, 8.8 μL of ddH_2_0. The RT-qPCR amplification conditions were listed as follows: UDG preprocessing at 50°C for 2 min; enzyme activation at 94°C for 10 min; pre-denaturation at 95°C for 3 min; followed by 40 cycles of denaturation at 95°C for 5 s, and annealing/extension at the corresponding temperature of each primer set for 30 s. The target specificity of each primer set was assessed by melting curve analysis, and the identity of all amplicons was verified by sequencing. The no-template controls and negative controls without reverse transcriptase were also included in all RT-qPCR runs. Each sample was run in triplicate. The relative mRNA expression levels of target genes were normalized to the two reference genes *GAPDH* and *β-ACTIN* using the 2^-ΔΔCT^ method (22). The primer pairs used in our RT-qPCR analysis are listed in Table 1.

**Table 1:** Primer sequences used for quantitative real-time reverse transcription PCR.

Gene name	Sequence (5′-3′)		Tm (°C)	Product length (bp)	GenBank accession number
*HSPA2*	F	GAACCCCACCAACACCATCT	56	108	XM_013183105.2
	R	TCATTCACCACACGGAAGGG			
*MYD88*	F	AGGGACGATCCATACGGGAA	58	154	XM_048062427.1
	R	CTGGCAAGACATCCCGATCA			
*NF-κB1*	F	TTCACATGGTGGTGATGGCA	57	106	XM_048066391.1
	R	TGCAATGCAGTTCGTCCAGA			
*NGF*	F	CCAAGTGCAGGGACCCTAAG	58	144	XM_013189623.2
	R	TTCGAATAAACCGCCAGGCT			
*PAK1*	F	AAACCTTTGCCTCCCAACCC	56	164	XM_048055172.1
	R	GTGACTGCATCAAACCCCAC			
*SPI1*	F	CATCTGGTGGGTGGACAAGG	58	96	XM_013193534.2
	R	CGATTGCCTTTCTGGATGCC			
*VEGFA*	F	TGAGGGCCTAGAATGTGTCC	57	84	XM_048080112.1
	R	ATGTGCTGACTCTGATGGGG			
*WNT5A*	F	TAGCCTGAAGACCTGTTGGC	60	89	XM_048047545.1
	R	TCATGGCAGCAGCACTATCG			
*β-ACTIN*	F	TGACAATGGCTCCGGTATGT	56	105	XM_013174886.1
	R	ACCATCACACCCTGATGTCTG			
*GAPDH*	F	AGCAACATCAAGTGGGCAGA	57	157	XM_013199522.2
	R	CACCCATCACGAACATGGGA			

HSPA2, heat shock protein family A member 2; MYD88, MYD88 innate immune signal transduction adaptor; NF-κB1, nuclear factor kappa B subunit 1; NGF, nerve growth factor; PAK1, p21 (RAC1) activated kinase 1; SPI1, Spi-1 proto-oncogene; VEGFA, vascular endothelial growth factor A; WNT5A, Wnt family member 5A; F, forward primer; and R, reverse primer.

### 2.5 Statistical analysis

All data were expressed as the mean ± SEM. The SAS 9.4 software (SAS Institute Inc., North Carolina, United States) was used for statistical analysis. Data were analyzed by two-way analysis of variance (ANOVA) using the general linear model (GLM) procedure in SAS 9.4 software, with the rearing system (MRS or CRS) and age (30 or 43 weeks of age) as the fixed factors. The Student’s *t*-test procedure was performed to assess the significance between two different groups. The Spearman correlation analysis was used to analyze the correlations between the splenic histomorphological characteristics and immune-related gene expression levels. A probability (*P*) value less than 0.01 was considered statistically extremely significant different, while a *p* value less than 0.05 was considered significant different. Both GraphPad Prism 8.0.2 (GraphPad Software, San Diego, CA, United States) and R 3.14 software (Lucent Technologies Inc., Milpitas, United States) were used to draw the figures. Results with *p* < 0.05 were considered statistically significant.

## 3 Results

### 3.1 Age-dependent effects of MRS and CRS on the weights and organ indexes of spleen and thymus among three goose breeds

**Figure 1:**
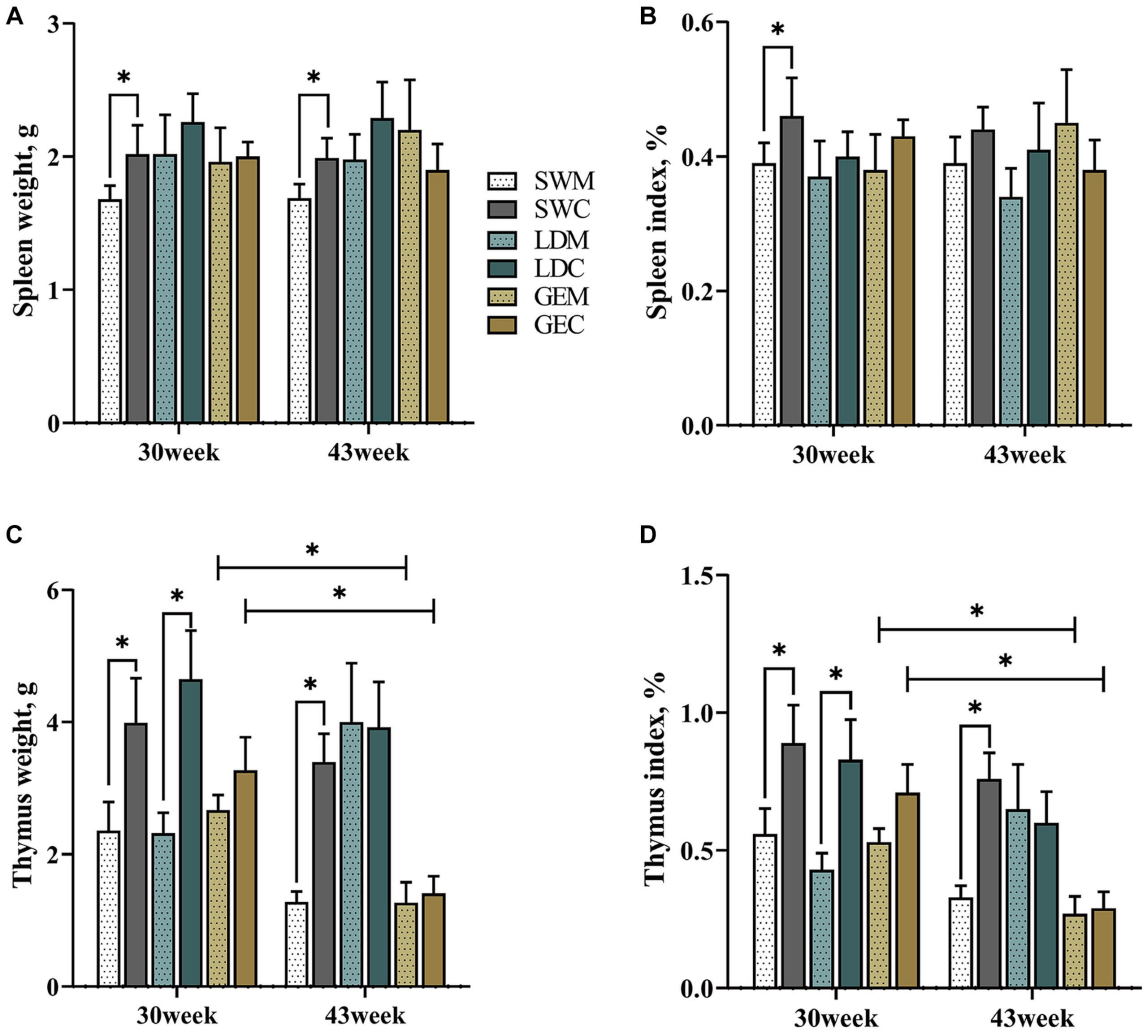
Comparison of the effects of MRS and CRS on immune organ development among three goose breeds. **(A)** Spleen weight. **(B)** Spleen organ index. **(C)** Thymus weight. **(D)** Thymus organ index. “*” indicates a significant difference between the two designated groups at the level of *p* < 0.05. Abbreviations: MRS, net-floor mixed rearing system; CRS, cage rearing system; SWM; Sichuan White goose under MRS; SWC, Sichuan White goose under CRS; LDM, Landes goose under MRS; LDC, Landes goose under CRS; GEM, Gang goose under MRS; and GEC, Gang goose under CRS.

As presented in Figure 1 and Supplementary Table 1, for SW, the weights and organ indexes of spleen and thymus were statistically significantly affected by the rearing system (*p* < 0.05). In detail, the splenic weight and organ index of SW were lower under MRS than under CRS at 30 or 43 weeks of age (*p* < 0.05). Similarly, the thymus weight and organ index of SW were lower under MRS than under CRS at both 30 and 43 weeks of age (*p* < 0.05), and those of LD were lower under MRS than under CRS at 30 weeks of age (*p* < 0.05). There was no significant difference in the splenic morphological parameters of GE between MRS and CRS at 30 and 43 weeks of age. Notably, the thymus weight and organ index generally decreased during the period from 30 to 40 weeks of age among three goose breeds, and were significantly affected by the age factor in GE (*p* < 0.05).

### 3.2 Age-dependent effects of MRS and CRS on the splenic histological paraments among three goose breeds

**Figure 2:**
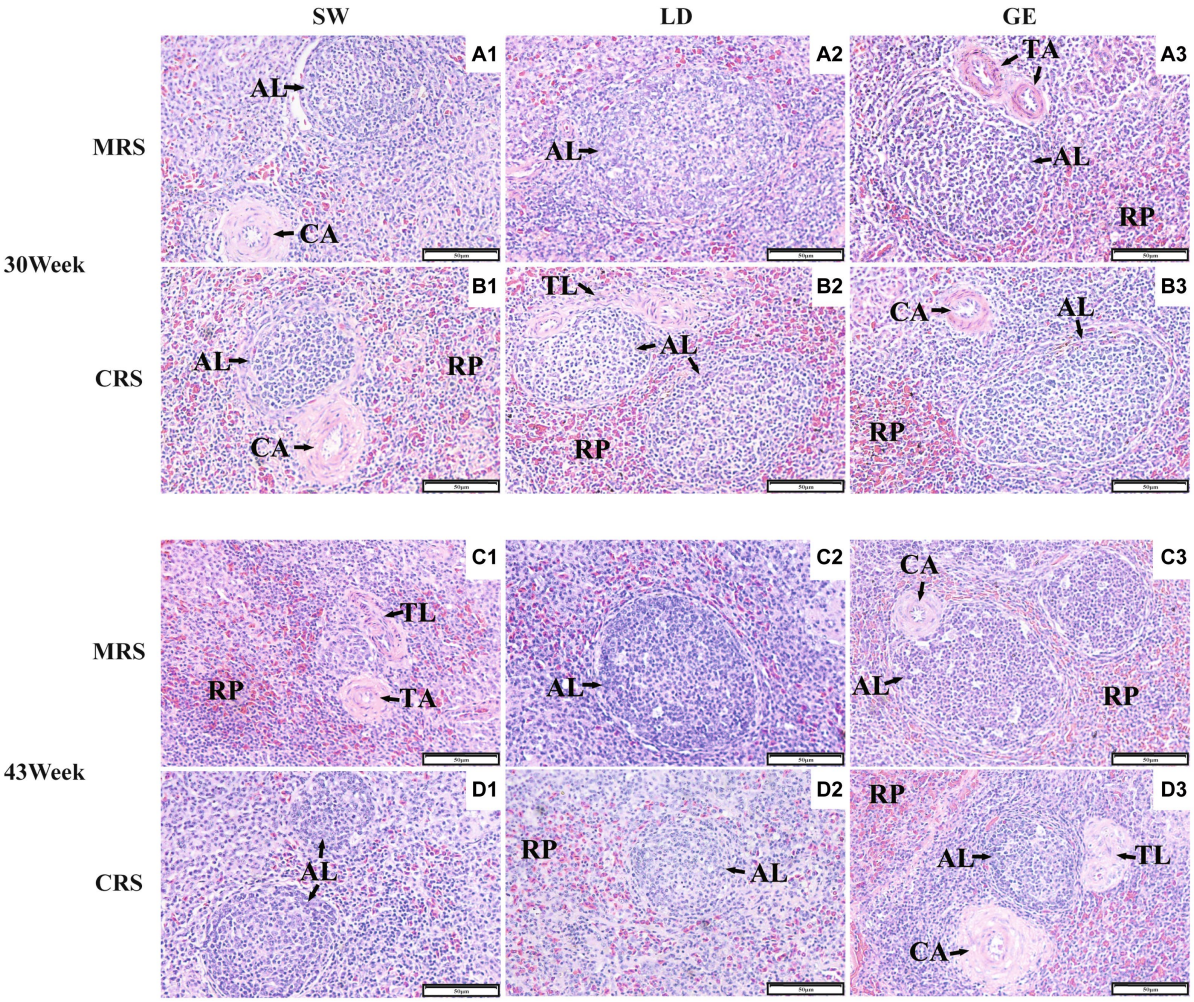
Histological observations of the spleens from three goose breeds under two different rearing systems. Representative histological images (200 ×) of the spleens from geese under MRS at 30 weeks of age **(A1–A3)**, under CRS at 30 weeks of age **(B1–B3)**, under MRS at 43 weeks of age **(C1–C3)**, and under CRS at 43 weeks of age **(D1–D3)**. The numbers 1, 2, and 3 represent the different breeds SW, LD, and GE. Abbreviations: MRS, net-floor mixed rearing system; CRS, cage rearing system; AL, splenic corpuscle; CA, central artery; TA, trabecular artery; TL, splenic trabecula; and RP, red pulp.

Next, we investigated the effects of MRS and CRS on the splenic histological paraments among three goose breeds. As shown in Figure 2, the goose spleen was histologically mainly composed of stroma and parenchyma. The stroma of the goose spleen was primarily composed of a reticular connective tissue network that provides support and containment for blood cells and immune cells, such as lymphocytes, macrophages, and dendritic cells. The parenchyma of the goose spleen was consisted of red and white pulp, without any definite demarcation of boundary. The red pulp was primarily composed of blood-filled sinusoids, while the white pulp is mainly populated by lymphoid tissue such as the splenic corpuscle.

**Figure 3:**
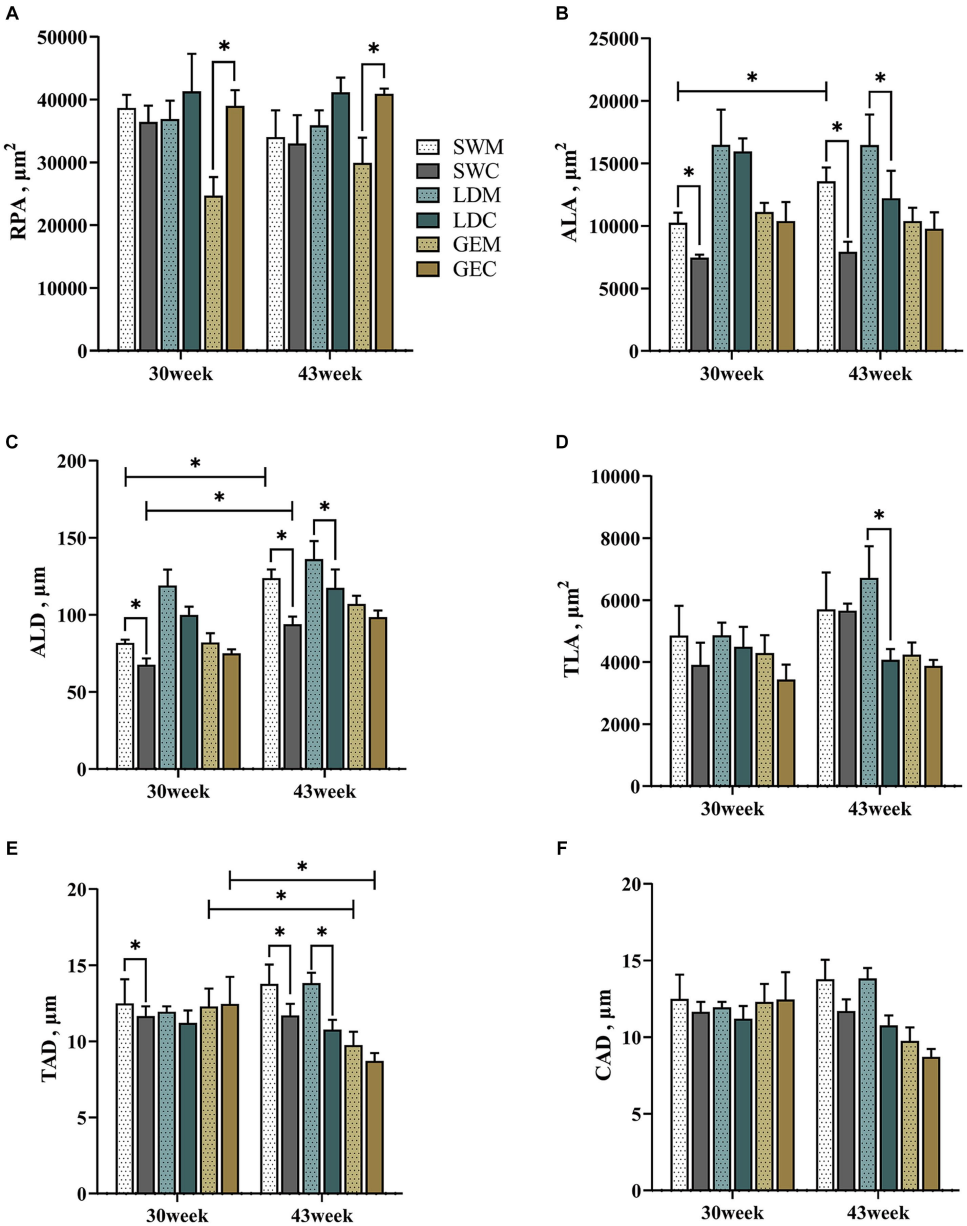
Comparison of the effects of MRS versus CRS on the spleen histological parameters among three goose breeds. **(A)** Red pulp area. **(B)** Splenic corpuscle area. **(C)** Splenic corpuscle diameter. **(D)** Splenic trabecula area. **(E)** Trabecular artery diameter. **(F)** Central artery diameter. “*” indicates a significant difference between the two designated two groups at the level of *p* < 0.05. Abbreviations: MRS, net-floor mixed rearing system; CRS, cage rearing system; SWM; Sichuan White goose under MRS; SWC, Sichuan White goose under CRS; LDM, Landes goose under MRS; LDC, Landes goose under CRS; GEM, Gang goose under MRS; and GEC, Gang goose under CRS.

Moreover, we analyzed differences in the measured splenic histological paraments between MRS and CRS. As shown in Figure 3 and Supplementary Table 2, the ALA, ALD, and TAD of SW were significantly affected by the rearing system (*p* < 0.001) and age (*p* < 0.05). For LD, the TLA and TAD were significantly affected by the rearing system (*p* < 0.05). For GE, the RPA was significantly affected by the rearing system (*p* < 0.001), while the TAD was significantly affected by age (*p* < 0.05). For SW and LD, some splenic histological paraments (including the ALA, ALD, and TAD) were larger under MRS than under CRS at 30 or 43 weeks of age (*p* < 0.05). For GE, the RPA was larger under CRS than under MRS (*p* < 0.05).

### 3.3 Age-dependent effects of MRS and CRS on immune-related gene expression profiles in spleens among three goose breeds

**Figure 4:**
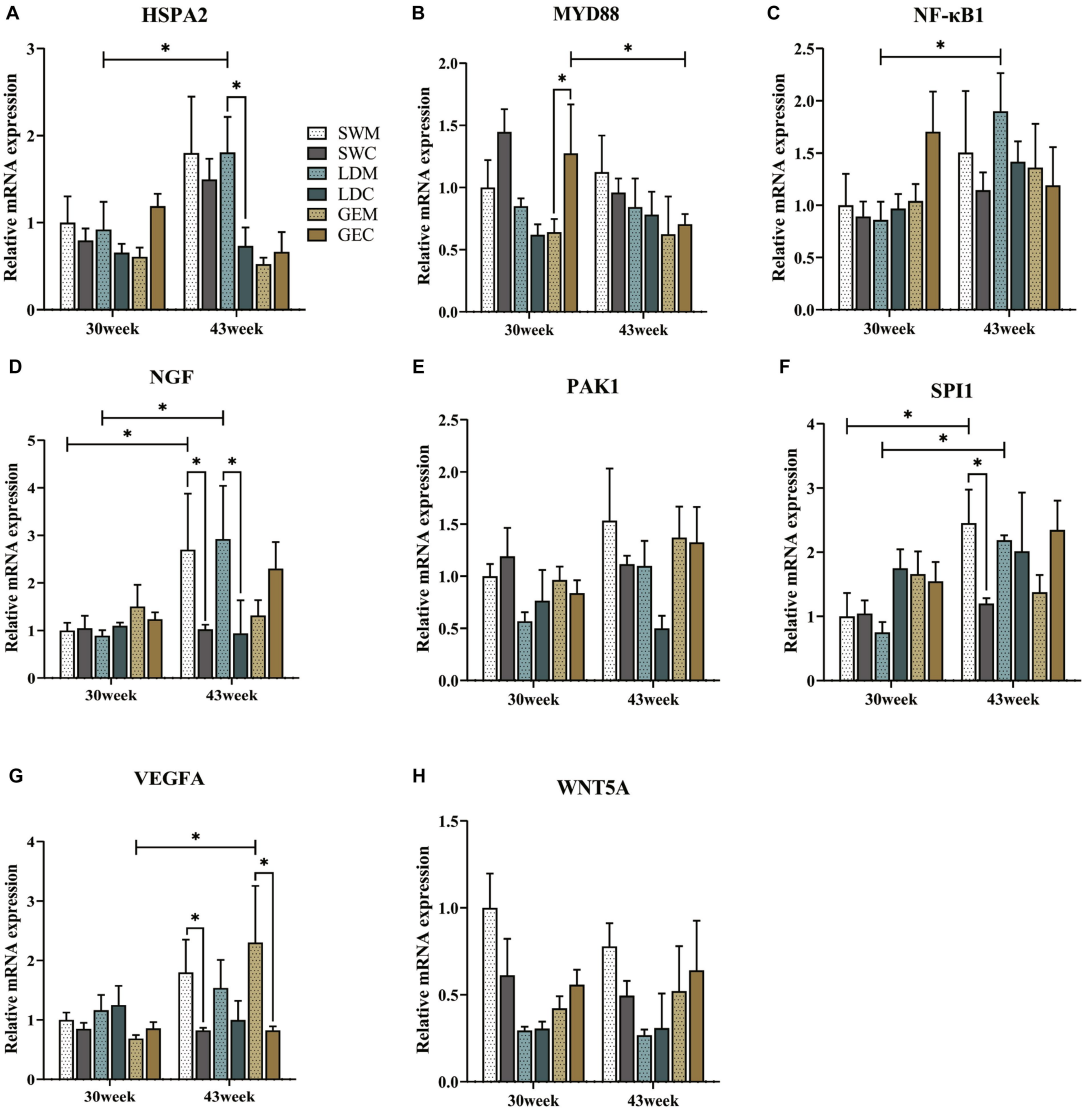
Comparison of the effects of MRS versus CRS on the spleen immune-related gene expression profiles among three goose breeds. The mRNA expression profiles of **(A)** *HSPA2*, **(B)** *MYD88*, **(C)** *NF-κB1*, **(D)** *NGF*, **(E)** *PAK1*, **(F)** *SPI1*, **(G)** *VEGFA*, and **(H)** *WNT5A* in spleens of three goose breeds under two different rearing systems. “*” indicates a significant difference between the two designated groups at the level of *p* < 0.05. Abbreviations: MRS, net-floor mixed rearing system; CRS, cage rearing system; SWM; Sichuan White goose under MRS; SWC, Sichuan White goose under CRS; LDM, Landes goose under MRS; LDC, Landes goose under CRS; GEM, Gang goose under MRS; and GEC, Gang goose under CRS.

We further explored differences in the mRNA expression levels of 8 immune-related genes, including *HSPA2*, *MYD88*, *NF-κB1*, *NGF*, *PAK1*, *SPI1*, *VEGFA*, and *WNT5A*, during the spleen development of geese reared under MRS and CRS. As shown in Figure 4, for SW, the mRNA levels of *NGF*, *SPI1*, and *VEGFA* were significantly higher under MRS than under CRS at 43 weeks of age (*p* < 0.05). Similarly, higher expression levels of *HSPA2* and *NGF* were observed in spleens of LD under MRS than those under CRS (*p* < 0.05). For GE, the *VEGFA* expression levels were significantly higher under MRS than under CRS at 43 weeks of age (*p* < 0.05), while the *MYD88* expression levels were significantly lower under MRS than under CRS at 30 weeks of age (*p* < 0.05). Furthermore, among three goose breeds, expression of *NF-κB1, PAK1*, and *WNT5A* tended to be higher under MRS when compared to CRS (0.05 < *p <* 0.1), with a more pronounced difference at 43 weeks of age.

### 3.4 Correlation analysis between the goose splenic histological paraments and gene expression profiles in response to different rearing systems

**Figure 5:**
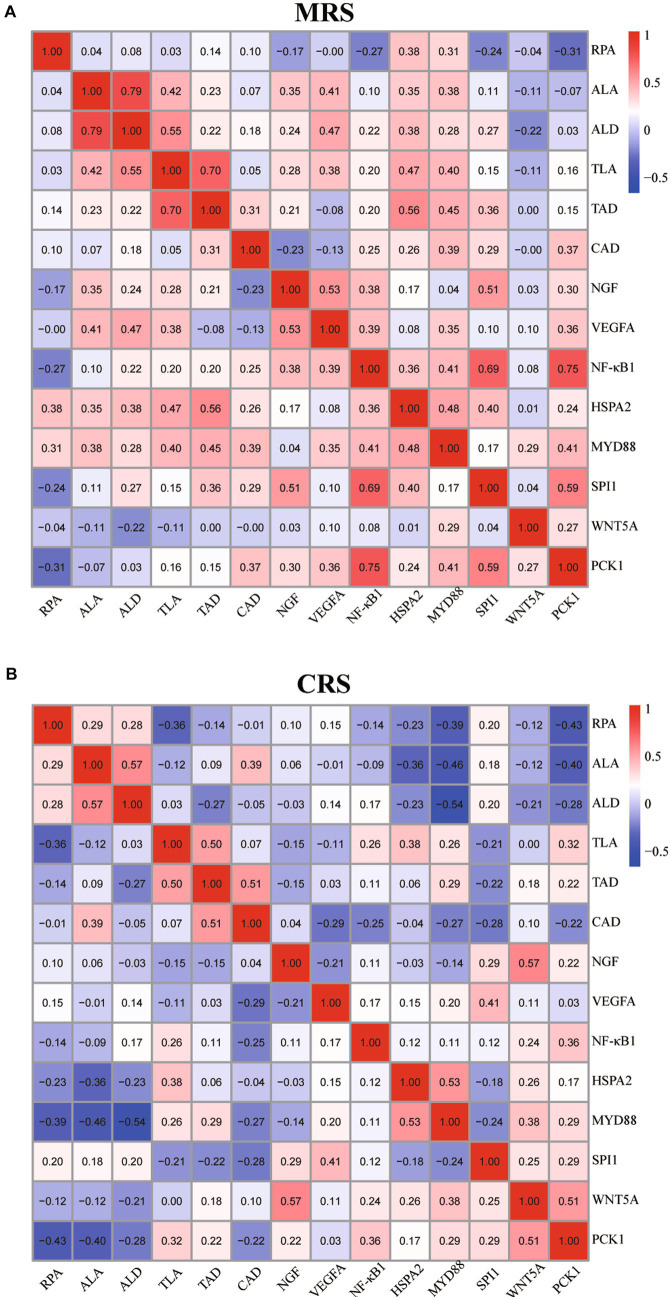
Correlation analysis of the goose spleen histological parameters and immune-related gene expression profiles under MRS **(A)** and CRS **(B)**. For both MRS and CRS, *n* = 24. The color represents the Spearman correlation coefficient. Abbreviations: MRS, net-floor mixed rearing system; CRS, cage rearing system.

Finally, we analyzed the correlations between the goose splenic histological paraments and immune-related genes expression profiles under different rearing systems. As shown in Figure 5 and Supplementary Figure 1, under CRS, some splenic histological paraments (including the RPA, ALA, ALD, and CAD) exhibited negative correlations with the mRNA (expression profiles of *HSPA2*, *MYD88*, *WNT5A*, and *PAK1*) while the TLA exhibited positive correlations with the expression profiles of *NF-κB1*, *HSPA2*, *MYD88*, and *PAK1*. By contrast, under MRS, there appeared to be more pronounced positive associations between the splenic histological paraments (including the ALA, ALD, TLA, and TAD) and the mRNA expression levels of *HSPA2*, *MYD88*, *NGF*, *SPI1*, and *VEGFA*.

## 4 Discussion

It has been widely reported that the rearing system has significant effects on the production and health of poultry. However, compared to chickens and ducks, little is known about the effects of dryland rearing systems on goose immune responses. Herein, we comprehensively analyzed the splenic morphological, histological, and molecular responses of three goose breeds to the two dryland rearing systems (MRS and CRS). Our morphological analysis revealed that the splenic weight and organ index of SW were higher under CRS than under MRS, while there were no significant differences in LD and GE. The thymus weight and organ index of three goose breeds, especially for GE, showed a decreasing tendency during development. In poultry, both spleen and thymus are vital for maintaining the body’s normal immune functions (23). It was reported that the chicken hens reared outdoor generally had a higher spleen weight and organ index than those reared indoor (24). The spleen and thymus weights of the ducks reared under NRS were higher than those reared under FRS (13). Therefore, it could be inferred that the dryland rearing system has significant effects on the goose splenic development, and the magnitude of these effects may depend on the breed, age, and their interaction.

Histological observations showed that the goose spleen can be divided into two main compartments, including the blood-containing red pulp and the white pulp that is full of lymphoid cells. The red pulp was reported to act as a blood filter by extracting aged, dead, or opsonized cells from the circulation (25). A previous study on the post-embryonic development of the duck blood-spleen barrier showed that the RPA gradually increased as the spleen developed, and so did the biological barrier functions (19). In the present study, a greater RPA was observed in spleens of GE reared under CRS than under MRS, which may lead to differences in their immune functions. The splenic corpuscle is where B cells gather and mature, and the periarterial lymphatic sheath around the central artery consists of matured T cells. Through these unique histological structures, the spleen can mount complex immune responses and clear pathogens from the blood effectively (26). A recent study on laying ducks showed that inflammation resulted in the proliferation of the splenic corpuscles and lymphocytes (27). Our results showed that for SW and LD, the ALA, ALD, and TAD were larger under MRS than under CRS at 30 or 43 weeks of age, implying that the geese reared under MRS might exhibit enhanced immune functions than those under CRS, particularly for SW and LD. It was noticeable that although the goose splenic morphological and histological paraments were differently affected by the two dryland rearing systems, the histological parameters could be better indicators of their immune functions.

To reveal the molecular mechanisms by which the rearing system regulates the goose spleen development, we further examined the expression of several key immune-related genes. Among them, *HSPA2*, a member of the heat-shock protein family, can protect cells from adverse stress such as oxidative stress, inflammation, and apoptosis. Meanwhile, *HSPA2* is involved in the activation of antigen-presenting cells, indirect presentation (or cross-priming), and translocation of NF-κB into the nuclei (28). The *NGF* can potentiate the activity of immune cells by activating the TLR4-NF-κB signaling and other anti-inflammatory pathways in humans, mice, and birds (29). It was reported that upregulated levels of *SPI1* could also activate the TLR4/NFκB signaling by binding to the *TLR4* promoter, which consequently aggravated cell apoptosis, inflammation, and immune cell recruitment (30). Besides, there is evidence that *VEGFA* was involved in the regulation of chronic inflammatory responses through the TLR4-NF-κB pathway (31). Notably, the NF-κB pathway is not only crucial for activating several pro-inflammatory cytokines in various cell types (32), but it also serves as a key regulator of innate and adaptive immunity, stress response, cell proliferation, and apoptosis (33). In this study, we observed that the mRNA expression levels of *HSPA2*, *NGF*, *SPI1*, and *VEGFA* were significantly differentially expressed in spleens of geese reared under MRS and CRS, suggesting that the rearing system may affect the goose spleen development and immune functions by regulating these key immune-related genes expression.

Furthermore, MYD88 is a key adaptor protein that mediates signaling pathways initiated by Toll-like receptors (TLRs) and the Interleukin-1 receptor (IL-1R) family. (34). Upon activation by microbial components or cytokines, MYD88 can recruit IRAK family kinases, leading to the activation of downstream signaling pathways, including the MAPK and NF-κB signaling pathways (35). Through these pathways, MYD88 is involved in the activation of immune cells in the spleen, such as macrophages, which promotes the production of inflammatory cytokines and the initiation of the immune response (36, 37). Our results showed that for GE, the *MYD88* mRNA expression levels were higher in spleens of geese reared under CRS than under MRS, suggesting a potential impact of the rearing system on the splenic immune response. Additionally, a previous study reported that overexpression of *PAK1* regulated the mouse immune responses against pathogens by promoting the inflammatory macrophage polarization via activation of the NF-*κ*B signaling (38). The *WNT5A*, which is recognized as a pro-inflammatory factor, has been shown to stimulate the pro-inflammatory cytokine release, angiogenesis, and lymphangiogenesis through the NF-*κ*B and MAPK signaling pathways (39, 40). In this study, there were no significant differences in the expression of *PAK1* and *WNT5A* in spleens of geese under MRS and CRS, implying that they might not directly mediate the effects of the rearing system on the goose spleen development.

The tight relationships among the splenic histomorphological characteristics, immune-related genes expression profiles, and the body’s immune functions have been widely reported in several domestic animals (13, 15). Similarly, our results showed that there appeared to be more pronounced positive associations between the splenic histological parameters (including the ALA, ALD, TLA, and TAD) and the mRNA expression levels of several key immune-related genes (*HSPA2*, *MYD88*, *NGF*, *SPI1*, and *VEGFA*) in geese reared under MRS than under CRS. Moreover, the age-dependent effects of the rearing system on the goose splenic histological parameters and immune-related genes expression profiles were found to be strongly synchronized, suggesting that these genes might play crucial roles in the regulation of goose immune functions. Their differential developmental expression profiles may be responsible for differences in the goose immunological responses to MRS and CRS.

## 5 Conclusion

In conclusion, we demonstrated the age-dependent differential effects of CRS and MRS on the splenic histomorphological characteristics and immune-related genes expression profiles among three goose breeds. The results showed that the geese reared under MRS might exhibit enhanced immune functions than those under CRS, particularly for SW and LD. Although these phenotypic differences are assumed to be associated with the age-dependent differential expression profiles of *HSPA2*, *MYD88*, *NGF*, *SPI1*, and *VEGFA* in the goose spleen, the underlying regulatory mechanisms await further investigations. It is noticeable that the different stocking density between CRS and MRS, which serves as one of the inherent properties of the dryland rearing systems, could at least by part, affect the goose immune response. Nevertheless, this study provides novel insights into the selection of suitable dryland rearing systems for improving goose immune status and the regulatory mechanisms of the rearing system on the goose spleen development.

## Data availability statement

The original contributions presented in the study are included in the article/Supplementary material, further inquiries can be directed to the corresponding author.

## Ethics statement

The animal studies were approved by Sichuan Agricultural University Animal Ethical and Welfare Committee. The studies were conducted in accordance with the local legislation and institutional requirements. Written informed consent was obtained from the owners for the participation of their animals in this study.

## Author contributions

QC: Conceptualization, Data curation, Formal analysis, Project administration, Writing – original draft. YS: Data curation, Methodology, Writing – original draft. ZH: Conceptualization, Formal analysis, Writing – original draft. GY: Investigation, Methodology, Writing – original draft. JuW: Resources, Software, Writing – original draft. XL: Methodology, Resources, Software, Writing – original draft. WW: Funding acquisition, Resources, Writing – review & editing. XY: Investigation, Writing – review & editing. JH: Resources, Writing – review & editing. HH: Project administration, Writing – review & editing. LL: Methodology, Writing – review & editing. JiW: Funding acquisition, Project administration, Validation, Writing – review & editing. SH: Funding acquisition, Project administration, Validation, Visualization, Writing – review & editing.
