# Supplementary material for: Effects of cage vs. net-floor mixed rearing system on goose spleen histomorphology and gene expression profiles
Source: Front Vet Sci. 2024 Feb 13;11:1335152. doi: 10.3389/fvets.2024.1335152 (PMC10896902; doi:10.3389/fvets.2024.1335152)
Supplement: Supplementary file 1 [file Table_1.docx]

**Supplementary Table 1.** Comparison of the effects of MRS versus CRS on immune organ development among three goose breeds.

| Items | | 30week | | 43week | | *P*-value | | |
| --- | --- | --- | --- | --- | --- | --- | --- | --- |
|  |  | MRS | CRS | MRS | CRS | RS | Age | RS × Age |
| SW | Spleen weight (g) | 1.68±0.10 | 2.02±0.22^#^ | 1.69±0.10 | 1.99±0.15^#^ | 0.008 | 0.810 | 0.492 |
|  | Spleen index (%) | 0.39±0.03 | 0.46±0.06^#^ | 0.39±0.04 | 0.44±0.03 | 0.024 | 0.668 | 0.389 |
|  | Thymus weight (g) | 2.36±0.43 | 3.99±0.68^#^ | 1.28±0.16 | 3.40±0.42^#^ | < 0.001 | 0.181 | 0.513 |
|  | Thymus index (%) | 0.56±0.09 | 0.89±0.14^#^ | 0.33±0.04 | 0.76±0.09^#^ | 0.001 | 0.198 | 0.524 |
| LD | Spleen weight (g) | 2.02±0.29 | 2.26±0.21 | 1.98±0.19 | 2.29±0.27 | 0.217 | 0.860 | 0.785 |
|  | Spleen index (%) | 0.37±0.05 | 0.40±0.04 | 0.34±0.04 | 0.41±0.07 | 0.293 | 0.580 | 0.577 |
|  | Thymus weight (g) | 2.32±0.31 | 4.65±0.74^#^ | 4.00±0.89 | 3.92±0.69 | < 0.001 | 0.959 | 0.216 |
|  | Thymus index (%) | 0.43±0.06 | 0.83±0.15^#^ | 0.65±0.16 | 0.60±0.11 | < 0.001 | 0.992 | 0.751 |
| GE | Spleen weight (g) | 1.96±0.26 | 2.00±0.11 | 2.20±0.38 | 1.90±0.20 | 0.527 | 0.742 | 0.609 |
|  | Spleen index (%) | 0.38±0.05 | 0.43±0.02 | 0.45±0.08 | 0.38±0.04 | 0.737 | 0.798 | 0.688 |
|  | Thymus weight (g) | 2.67±0.23* | 3.27±0.50* | 1.27±0.31 | 1.41±0.26 | 0.295 | < 0.001 | 0.438 |
|  | Thymus index (%) | 0.53±0.05* | 0.71±0.10* | 0.27±0.06 | 0.29±0.06 | 0.218 | < 0.001 | 0.458 |

* indicates a significant difference in the same breed under a same rearing system between different weeks of age at the level of *P* < 0.05.

^#^ indicates a significant difference in the same breed with a same age between different rearing systems at the level of *P* < 0.05.

Abbreviations: SW, Sichuan White goose; LD, Landes goose; GE, Gang goose; MRS, net-floor mixed rearing system; CRS, cage rearing system; and RS, rearing system.
