# Supplementary material for: Effects of cage vs. net-floor mixed rearing system on goose spleen histomorphology and gene expression profiles
Source: Front Vet Sci. 2024 Feb 13;11:1335152. doi: 10.3389/fvets.2024.1335152 (PMC10896902; doi:10.3389/fvets.2024.1335152)
Supplement: Supplementary file 2 [file Table_2.docx]

**Supplementary Table 2.** Comparison of the effects of MRS versus CRS on the spleen histological parameters among three goose breeds.

| Items | | 30week | | 43week | | *P*-value | | |
| --- | --- | --- | --- | --- | --- | --- | --- | --- |
|  |  | MRS | CRS | MRS | CRS | RS | Age | RS × Age |
| SW | RPA (μm^2^) | 38686.77±2075.14 | 36448.62±2595.26 | 34060.51±4215.60 | 33028.91±4488.06 | 0.649 | 0.273 | 0.866 |
|  | ALA (μm^2^) | 10254.51±793.48^#^ | 7468.74±225.93 | 13561.75±1113.81^#^* | 7937.79±801.03 | < 0.001 | 0.036 | 0.102 |
|  | ALD (μm) | 81.79±2.11^#^ | 67.63±4.12 | 123.79±5.63^#^* | 93.89±4.89* | < 0.001 | < 0.001 | 0.098 |
|  | TLA (μm^2^) | 4859.60±956.92 | 3915.99±711.71 | 5703.91±1187.99 | 5660.89±223.85 | 0.572 | 0.153 | 0.606 |
|  | TAD (μm) | 12.50±1.58^#^ | 11.65±0.64 | 13.78±1.26^#^ | 11.70±0.76 | < 0.001 | 0.025 | 0.444 |
|  | CAD (μm) | 17.20±1.47 | 15.41±1.36 | 20.41±2.28 | 18.72±2.15 | 0.367 | 0.105 | 0.977 |
| LD | RPA (μm^2^) | 36900.29±2938.96 | 41312.95±5977.15 | 35924.88±2366.31 | 41175.91±2333.12 | 0.219 | 0.884 | 0.912 |
|  | ALA (μm^2^) | 16489.12±2818.69 | 15966.10±1029.79 | 16470.88±2441.14^#^ | 12203.41±2214.69 | 0.304 | 0.413 | 0.417 |
|  | ALD (μm) | 118.99±10.34 | 99.95±5.25 | 136.17±11.67^#^ | 117.54±11.88 | 0.088 | 0.112 | 0.984 |
|  | TLA (μm^2^) | 4865.13±409.15 | 4498.89±642.89 | 6722.26±1018.23^#^ | 4075.11±347.55 | 0.041 | 0.298 | 0.109 |
|  | TAD (μm) | 11.94±0.45 | 11.21±0.81 | 13.83±0.68^#^ | 10.76±0.65 | 0.012 | 0.286 | 0.095 |
|  | CAD (μm) | 17.31±0.91 | 17.14±0.87 | 18.93±2.89 | 14.78±2.29 | 0.379 | 0.487 | 0.438 |
| GE | RPA (μm^2^) | 24719.89±2943.42 | 39018.13±2473.65^#^ | 29925.99±4000.79 | 40948.05±808.94^#^ | < 0.001 | 0.227 | 0.570 |
|  | ALA (μm^2^) | 11283.53±815.30 | 10388.12±1516.37 | 10376.98±1074.40 | 9767.27±1324.01 | 0.175 | 0.141 | 0.189 |
|  | ALD (μm) | 82.08±5.96 | 75.01±2.62 | 107.10±5.30 | 98.42±4.42 | 0.434 | 0.621 | 0.055 |
|  | TLA (μm^2^) | 4292.26±572.40 | 3441.58±480.02 | 4244.93±384.98 | 3878.28±192.10 | 0.332 | 0.209 | 0.092 |
|  | TAD (μm) | 12.29±1.17* | 12.46±1.78* | 9.76±0.87 | 8.72±0.51 | 0.718 | 0.021 | 0.617 |
|  | CAD (μm) | 16.85±2.35 | 17.47±1.54 | 15.00±1.28 | 13.02±1.44 | 0.699 | 0.089 | 0.460 |

* indicates a significant difference in the same breed under a same rearing system between different weeks of age at the level of *P* < 0.05.

^#^ indicates a significant difference in the same breed with a same age between different rearing systems at the level of *P* < 0.05.

Abbreviations: SW, Sichuan white goose; LD, Landes goose; GE, Gang goose; MRS, net-floor mixed rearing system; CRS, cage rearing system; RS, rearing system; RPA, red pulp area; ALA, splenic corpuscle area; ALD, splenic corpuscle diameter; TLA, splenic trabecula area; TAD, trabecular artery diameter; and CAD, central artery diameter.
