# Supplementary figures and images for: Effects of cage vs. net-floor mixed rearing system on goose spleen histomorphology and gene expression profiles
Source: Front Vet Sci. 2024 Feb 13;11:1335152. doi: 10.3389/fvets.2024.1335152 (PMC10896902; doi:10.3389/fvets.2024.1335152)

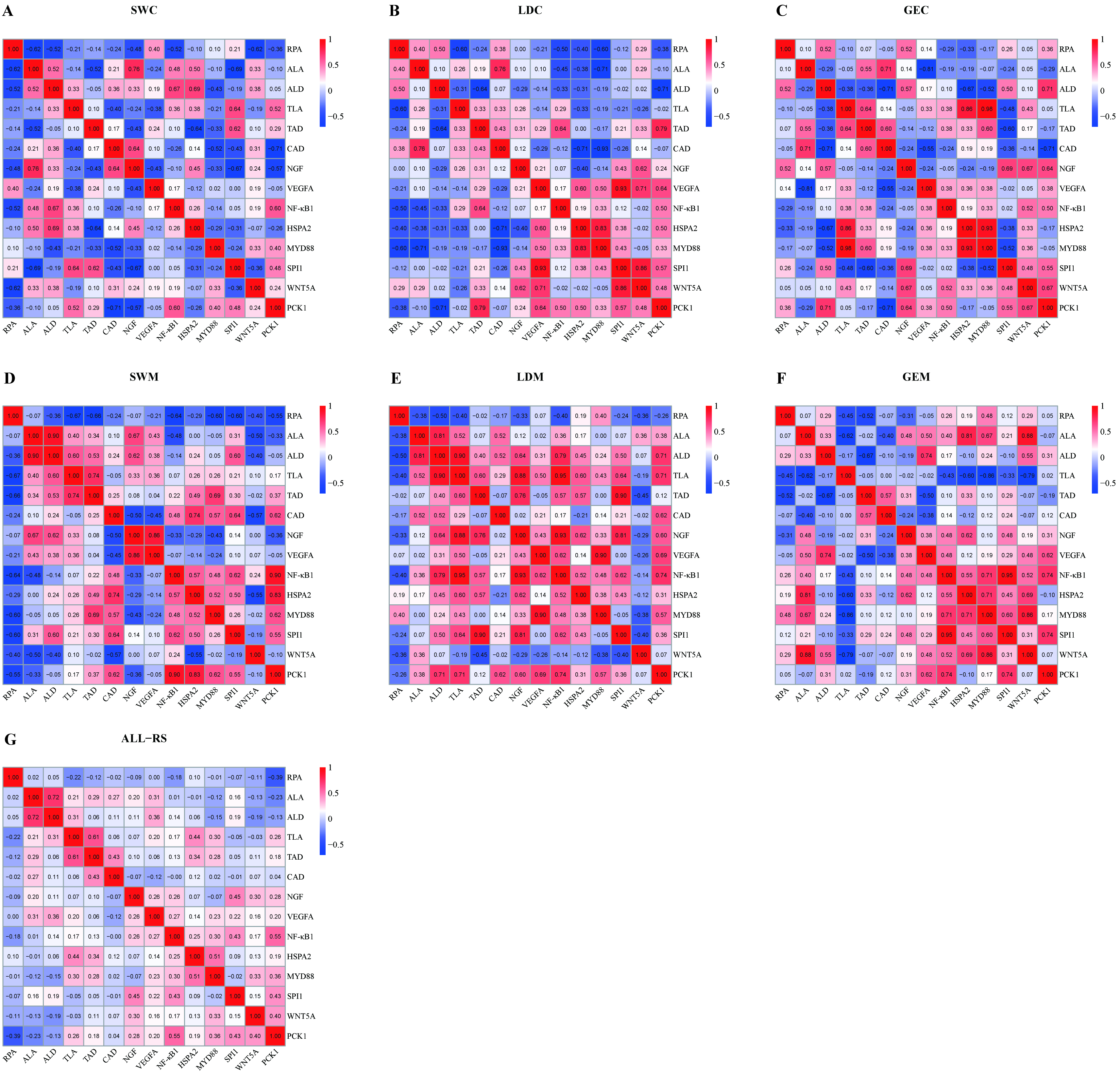

Supplement: Supplementary file 3 [file Image_1.tif]
